# Antifungal prophylaxis in chemotherapy-associated neutropenia: a retrospective, observational study

**DOI:** 10.1186/1471-2334-7-70

**Published:** 2007-07-02

**Authors:** Amy Riedel, Lan Choe, John Inciardi, Courtney Yuen, Thomas Martin, B Joseph Guglielmo

**Affiliations:** 1 Department of Clinical Pharmacy, School of Pharmacy, University of California San Francisco, San Francisco, USA; 2 Division of Hematology/Oncology, Department of Medicine, University of California-San Francisco, San Francisco, USA

## Abstract

**Background:**

In August 2002, the antifungal prophylaxis algorithm for neutropenic hematology/oncology (NHO) patients at the Medical Center was changed from conventional amphotericin (AMB) to an azole (AZ) based regimen (fluconazole [FLU] in low-risk and voriconazole [VOR] in high-risk patients). The aim of our study was to compare outcomes associated with the two regimens, including breakthrough fungal infection, adverse drug events, and costs.

**Methods:**

Adult, non-febrile, NHO patients who received prophylactic AMB from 8/01/01-7/30/02 or AZ from 8/01/02-7/30/03 were retrospectively evaluated.

**Results:**

A total of 370 patients (AMB: n = 181; AZ: n = 216) associated with 580 hospitalizations (AMB: n = 259; AZ: n = 321) were included. The incidence of probable/definite  breakthrough Aspergillus infections was similar among regimens (AMB:  1.9% vs AZ: 0.6%; p=0.19). A greater incidence of mild/moderate (24.7% vs. 5.3%; p < 0.0001) and severe renal dysfunction (13.5% vs. 4.4%; p < 0.0012) was observed with AMB. In contrast, patients treated with VOR were found to have an increased rate of severe hepatic toxicity (32.5%) compared with patients treated with either AMB (22.6%) or FLU (21.4%) (p = 0.05). While the AZ period was associated with a >$9,000 increase in mean total costs/hospitalization, the mean acquisition cost associated with AZ was only $947/hospitalization more than AMB.

**Conclusion:**

While an AZ-based regimen is associated with increased cost, the reduced rate of nephrotoxicity and availability of oral dosage forms, suggests that azoles be used preferentially over AMB. However, an increased rate of severe hepatic toxicity may be associated with VOR.

## Background

Invasive fungal infection due to *Candida *and *Aspergillus *is associated with increased morbidity and mortality in neutropenic hematology and oncology (NHO) patients. Amphotericin B (AMB) is a broad-spectrum antifungal agent used for prophylaxis of fungal infections in neutropenic hematology/oncology patients. However, conventional AMB is associated with an increased risk of infusion-related reactions and renal toxicity. Prospective, randomized, controlled trials have demonstrated that fluconazole (FLU) and voriconazole (VOR) are safe and effective alternatives to AMB in febrile, neutropenic patients and fluconazole has been found to be an effective prophylactic agent in neutropenia [1-4]. The Centers for Disease Control, Infectious Disease Society of America, and American Society of Blood and Bone Marrow Transplantation currently recommend FLU for the prevention of candidiasis for patients undergoing hematopoietic stem cell transplantation [5]. Of concern, FLU has inadequate activity versus *Aspergillus spp*. Although VOR has adequate activity versus *Aspergillus spp*, it is currently not approved by the Food and Drug Administration for use in febrile neutropenia. However, Walsh et al. (2002) demonstrated that VOR was superior to liposomal AMB in reducing documented breakthrough fungal infections (p = 0.02), severe infusion-related toxicity (p < 0.01), and renal toxicity (p < 0.001) in patients with febrile neutropenia [2]. Based on the results of this study, in August 2002, the University of California at San Francisco (UCSF) antifungal prophylaxis regimen was changed from conventional AMB to an azole-based regimen that included FLU and VOR. The objectives of this study were to compare the rate of breakthrough fungal infection, adverse drug events and costs associated with these prophylactic regimens.

## Methods

### Patients

#### Eligibility criteria

The UCSF Committee on Human Research approved the study. All patients were treated at the UCSF Medical Center. Adult neutropenic (defined as an absolute neutrophil count < 500 cells/mm^3^) hematology/oncology patients who received conventional AMB prophylaxis from August 1, 2001 through July 30, 2002, or who received FLU or VOR prophylaxis from August 1, 2002 to July 30, 2003, were included. During the AZ period, low-risk patients, defined as leukemics or those receiving autologous transplant, received prophylaxis with FLU. High-risk patients, defined as those receiving allogeneic stem cell transplants, received prophylaxis with VOR. Patients were excluded if they received azoles or AMB for reasons other than prophylaxis.

### Administration of Study Medications

In accordance with the UCSF antifungal prophylaxis algorithm, from August 1, 2001 through July 30, 2002, NHO patients received conventional intravenous AMB 0.3 mg/kg/day initially titrated to 0.7 mg/kg/day as tolerated. From August 1, 2002 to July 30, 2003, low-risk NHO patients received FLU 400 mg by mouth daily and high-risk patients received VOR 200 mg by mouth twice daily. If unable to tolerate oral medications, patients could receive either FLU (400 mg/daily) or VOR 200 mg every 12 hrs intravenously.

### Study Design

This investigation was a retrospective, observational, cohort study. Non-blinded reviews of patients' electronic files at UCSF, the pharmacy acquisition cost database, and the medical center cost accounting system were conducted. Collected information included: gender, age, diagnosis, cost of hospitalization (including pharmacy, chemistry laboratory, and hemodialysis), length of hospital stay, renal toxicity, hepatotoxicity, breakthrough fungal infections, hemodialysis (HD) requirements, reason for HD, and modification of antifungal prophylaxis. Duration of antifungal prophylaxis was calculated by the number of days from the start of therapy to discharge.

Diagnoses were condensed into the following groups: Hodgkin's disease, non-Hodgkin's lymphoma (including Burkitt's lymphoma, mantle cell lymphoma, T cell lymphoma, angioimmunoblastic lymphadenopathy, aggressive B-cell lymphoma, natural killer cell lymphoma, and cutaneous T-cell anaplastic lymphoma), myeloid neoplasms (including acute myelogenous leukemia, chronic myelogenous leukemia, myelofibrosis, and myelodysplastic syndrome), lymphocytic leukemia (including acute and chronic lymphocytic leukemia), multiple myeloma, and other hematologic disorders/malignancies (including anaplastic plasmacytoma, aplastic anemia, amyloidosis, and light chain deposition disease).

#### Efficacy assessment

The definitions of possible, probable, and definite breakthrough fungal infections were based upon clinical consensus guidelines on opportunistic fungal infections in cancer patients and stem cell recipients [6].

#### Safety assessment

Renal and hepatic toxicity were defined as per previous published trials of AMB, FLU, and VOR [2-4,7,8]. Mild to moderate renal toxicity was defined as >0.4 mg/dL increase in serum creatinine (Scr). Severe renal dysfunction was defined as at least a doubling of the Scr level (or an increase of at least 1 mg/dL if the base line was above upper normal limits).

Hospitalizations associated with acute HD or continuous veno-venohemofiltration (CVVH) were identified using billing codes. The patient's electronic files were further reviewed to determine etiology of renal failure and confirm the mode of dialysis.

Mild to moderate hepatic toxicity was defined as a tripling of the baseline value of serum aspartate aminotransferase (AST), or serum alanine aminotransferase (ALT), or serum alkaline phosphatase (Alk Phos) or a 1.5-fold increase in serum total bilirubin during therapy. Severe hepatic toxicity was defined as at least a five-fold increase in the baseline value of AST, ALT, or Alk Phos or a three-fold increase in serum total bilirubin during therapy.

#### Cost Analysis

Total hospital costs represent the UCSF cost of providing service and do not reflect charges or reimbursement. TSI (Transition System Inc., Eclipsys) was used to determine total, pharmacy and laboratory costs for all patients.

### Statistical Analysis

Due to multiple hospitalizations for patients, continuous variables (age, length of stay, duration of therapy, baseline and peak lab values [Scr, AST, ALT, Alk Phos, T bili], costs) were analyzed using mixed effects logistic regression. Some of these variables (length of stay, duration of therapy, costs) were then log transformed to improve the distribution. These models controlled for the number of hospitalizations and include fixed effects for each medication and for each period.

Similarly, discrete variables (rates of infection, renal toxicity, hepatic toxicity) were analyzed using random effects logistic regression. These models controlled for the number of hospitalizations and include fixed effects for each medication and for each period.

Multi-variable analyses were conducted for the outcome of total costs using mixed effects regression models that controlled for age, diagnoses, therapy (allogeneic transplant, autologous transplant, chemotherapy, other diagnoses), and duration of antifungal prophylaxis. The cost variables were then log transformed to improve the distribution. Multi-variable analyses were also conducted for the outcome of all fungal infection using random effects regression models that controlled for age, diagnoses, therapy (allogeneic transplant, autologous transplant, chemotherapy, other diagnoses), and duration of antifungal prophylaxis. These models include fixed effects for each medication and for each period.

Statistical significance was defined as a P value of < 0.05.

## Results

### Patient Characteristics

A total of 370 patients associated with 580 hospitalizations were included in the analysis (Table 1). During the AZ period, FLU (low-risk patients) was used in 237 (74%) and VOR (high-risk patients) for 84 (26%) of the hospitalizations. During the AMB period, the majority of patients were hospitalized either once (68.5%) or twice (21.5%). Similarly, in the AZ period, most patients were hospitalized once (FLU 74.3%; VOR 78.1%) or twice (FLU 18.1%; VOR 12.5%). Twenty-seven patients were treated during both periods. During the AZ period, 19 patients were treated with both FLU and VOR during multiple hospitalizations.

**Table 1:** Patient Characteristics

	AMB Period vs. AZ Period			
	AMB	AZ	Odds Ratio^1^	p-value
No. of Hospitalizations	259	321		
No. of Patients	181	216		
Mean Age^2^	49.6 yrs (19 – 86 yrs)^3^	50.3 yrs (18 – 83 yrs)		<0.001^4^
Gender	65% male	55% male	OR = 0.00	0.0009^5^
Diagnoses				
NHL	74 (29%)	102 (32%)	OR = 0.96	0.97
Hodgkin's Disease	11 (4%)	19 (6%)	OR = 1.38	0.85
Myeloid Neoplasms	96 (37%)	111 (35%)	OR = 0.56	0.6
Lymphocytic Leukemia	46 (18%)	40 (12%)	OR = 0.58	0.66
Multiple Myeloma	25 (10%)	39 (12%)	OR = 1.16	0.9
Other	7 (3%)	10 (3%)	OR = 6.45	0.25
Therapy				
Mini-transplant	14 (8%)	15 (5%)	OR = 0.98	0.96^5^
Allo-transplant	3 (2%)	23 (7%)	OR = 2.62	0.22^5^
Auto-transplant	39 (22%)	42 (13%)	OR = 0.63	0.03^5^
Chemotherapy	113 (62%)	136 (42%)	OR = 1.16	0.55^5^
Other	11(6%)	19 (6%)	OR = 1.24	0.59^5^
Twin-transplant	1 (0.6%)	0		
Mean Length of Stay^2^	20.9 days (19–23 days)	19.2 days (17.5–21.1 days)		0.21^4^
Mean Duration of therapy^2^	19.8 days (17.9–21.8 days)	16.5 days (15–18.1 days)		0.009^4^

^1^Odds ratios are from random effects regression models that controlled for the number of hospitalizations. The reported odds ratios are the odds of having the individual variables in the AZ period relative to the AMB period

^2^Reported means are least squared means from mixed effects regression models that controlled for the number of hospitalizations

^3^age range is the observed age range of the patients in each period

^4^p-values are from mixed effects logistic regression models that controlled for the number of hospitalization

^5^p-values are from random effects logistic regression models that controlled for the number of hospitalization

Note: AMB = amphotericin; AZ = Azole; FLU = fluconazole; VOR = voriconazole; NHL = non-Hodgkin's lymphoma; OR = odds ratio; Allo-transplant = allogeneic stem cell transplant; auto-transplant = autologous stem cell transplant

Patients in the AZ period were older than patients in the AMB period (50.3 vs. 49.6 years of age, respectively; 95% Confidence Interval [CI] -0.85 – -0.65, standard error [SE] = 0.052, p < 0.001). The admitting diagnoses were similar between periods; the majority of diagnoses were myelogenous neoplasms, non-Hodgkin's lymphoma, lymphocytic leukemia, and multiple myeloma. Significantly fewer patients in the AZ period underwent auto-transplant (13% vs. 22%, OR = 0.63, 95% CI 0.42–0.95, p = 0.03). Otherwise, treatments (mini-transplant, allo-transplant, chemotherapy, etc) were similar between periods (p > 0.2). The following baseline lab values were similar between the two periods: Scr (AZ 1.01 vs. AMB 0.97; 95% CI 0.9 – 1.03, SE = 0.035, p = 0.28), AST (AZ 28.65 vs. AMB 26.32; 95% CI 0.84 – 1.00, SE = 0.05 p = 0.08), ALT (AZ 28.73 vs. AMB 27.85; 95% CI 0.84 – 1.12, SE = 0.07, p = 0.67), Alk Phos (AZ 79.76 vs AMB 77.27; 95% CI 0.88 – 1.07, SE = 0.05, p = 0.51), and Tbili (AZ 0.74 vs. AMB 0.74; 95% CI 0.9 – 1.11, SE = 0.05, p = 0.98)

Patients in the AMB period received antifungal prophylaxis significantly earlier than patients in the AZ period (1.8 vs. 3.9 days from admission to start of therapy; 95% CI – 2.87 – -1.37, SE = 0.38, p < 0.001). The mean lengths of stay were similar (19.2 days in the AZ period and 20.9 days in the AMB period; 95% CI = 8.9 – 17.4, SE = 1.16, p = 0.21). Duration of antifungal prophylaxis was significantly longer during the AMB period (19.8 days vs. 16.5 days; 95% CI 11.22 – 23.44, SE = 1.17, p = 0.009).

### Breakthrough Fungal Infections

Overall, the incidence of breakthrough fungal infections was similar in the AZ and AMB periods (13.7% vs. 10.4% respectively; OR = 1.38, 95% CI 0.82 – 2.34, p = 0.23) (Table 2). After multi-variable analyses controlling for age, diagnoses, therapy (allogeneic transplant, autologous transplant, chemotherapy, other diagnoses), and duration of antifungal prophylaxis, the rate of breakthrough fungal infection was similar during both the AZ and AMB period (OR = 1.53, 95% CI 0.852 – 2.761, p = 0.15).

**Table 2:** Breakthrough fungal infections

	AMB Period vs. AZ Period			
	AMB (n = 259)	AZ (n = 321)	Odds Ratio^1^	p-value^2^
Any Fungal Infection	27 (10.4%)	44 (13.7%)	OR = 1.38	0.23
Possible	18 (6.9%)	37 (11.5%)		
Probable/Definite	9 (3.5%)	7 (2.2%)		
Aspergillus	5 (1.9%)	2 (0.6%)	OR = 0.33	0.19
Candida	4 (1.5%)	5 (1.6%)	OR = 0.97	0.97

^1^Odds ratios are from random effects regression models that controlled for the number of hospitalizations. The reported odds ratios are the odds of having a breakthrough infection in the AZ period relative to the AMB period

^2^p-values are from random effects logistic regression models that controlled for the number of hospitalization

*Two patients in the FLU group had both Aspergillus and Candida infections in the same hospitalization

*Of the two patients in the AZ group who had probable/definite breakthrough Aspergillus infection, one patient received FLU and the other received VOR

Note: AMB = amphotericin; AZ = Azole; FLU = fluconazole; VOR = voriconazole

While not statistically significant, the rate of probable and definite breakthrough fungal infections, including that due to Aspergillus, was less during the AZ period (AZ 0.6% vs. AMB 1.9%; OR = 0.33, 95% CI 0.06 – 1.73, p = 0.19).

Three of the probable/definite Aspergillus infections in the AMB period were fatal; neither of the probable/definite Aspergillus infections in the AZ period was fatal. Of the two patients in the AZ period who developed a probable/definite Aspergillus infection, one received FLU and the other received VOR.

While infrequent, the rate of probable and definite Candida infections in the AMB and AZ periods was similar between groups (1.5% vs. 1.6%, respectively; OR = 0.97, 95% CI 0.25 – 3.76, p = 0.97). During the AMB period, probable breakthrough Candida infections included hepatosplenic candidiasis (n = 1), esophagitis (n = 1), and thrush (n = 2). In the AZ period, two cases of probable Candida infections (oral candidiasis and one case of splenic candidiasis were observed. The patient with probable splenic candidiasis also had possible Aspergillus pulmonary disease. Three cases of definite disseminated fungal infections (*Candida kruseii *[n = 1] and *Candida glabrata *[n = 2]) were observed during the AZ period. One patient with *Candida glabrata *sepsis had a concomitant possible Aspergillus pneumonia. Of those patients with breakthrough Candida fungal infection, all received prophylaxis with FLU.  No cases of breakthrough  candidal infection were observed with VOR prophylaxis.

### Renal Dysfunction

Fewer hospitalizations in the AZ period were associated with mild/moderate (AZ 5.3% vs. AMB 24.7%; OR = 0.18, 95% CI 0.11 – 0.3, p < 0.0001) and severe renal dysfunction (AZ 4.4% vs. AMB 13.5%; OR = 0.29, 95% CI 0.14 – 0.64, p < 0.0012). Hemodialysis requirements were infrequent during both time periods (AMB: 2.3%; AZ: 4%). Eleven of 19 patients (58%) requiring HD were found to be HD-dependent prior to admission. Two patients in the AMB period and four patients in the AZ period developed new onset renal failure requiring HD. Notably, one patient in the AZ period was switched to lipid-based AMB and subsequently developed acute renal failure requiring HD.

### Hepatic Toxicity

Serum AST, ALT, Alk Phos and T bili levels were available for 540 hospitalizations (AMB = 243; FLU = 217; VOR = 80). The AZ period was associated with a lower incidence of mild/moderate hepatic toxicity (AZ 33.7% vs. AMB 48.6%; OR = 0.58, 95% CI 0.29 – 1.05, p = 0.03). The rate of severe hepatic toxicity did not differ between periods (AZ 21.4% vs. AMB 25.3%; OR = 1.23, 95% CI 0.81 – 2.03, p = 0.28). However, patients receiving VOR prophylaxis had a greater incidence of severe hepatic toxicity when compared with those patients treated with either AMB or FLU (32.5% vs. 21.4% vs. 22.6%, respectively; OR = 1.91, 95% CI 1 – 3.64, p = 0.0507). Patients receiving VOR (n = 80) experienced a higher average peak AST (VOR 56 vs. FLU 39, 95% CI 1.2 – 1.8, SE = 0.1, p < 0.001; VOR 56 vs. AMB 39, 95% CI 1.2 – 1.7, SE = 0.1, p < 0.001) and ALT (VOR 70 vs. FLU 44, 95% CI 1.3 – 2.0, SE = 0.11, p < 0.001; VOR 70 vs. AMB 50, 95% CI 1.1 – 1.8, SE = 0.11, p < 0.001).

### Modification of Antifungal Prophylaxis

Modification of antifungal therapy took place more frequently during the AZ period (AZ 13.1% vs. 23% AMB; OR = 2.19, 95% CI = 1.3 – 3.69, p = 0.003). Modification was most commonly associated with initial receipt of FLU and resulted in a switch to VOR due to possible, probable or definite breakthrough fungal infection (n = 25), development of new neutropenic fevers (n = 36), and initiation of high dose steroids (n = 1). There was no difference in the rate of discontinuation between AMB and VOR (13.1% vs. 11.9%, respectively p = 0.76). VOR prophylaxis was modified in response to elevated LFTs (n = 8), QTC prolongation (n = 1), and for unknown reasons (n = 1). Modification of AMB to lipid-based AMB, itraconazole, FLU or VOR took place in response to an increasing Scr (n = 18), anaphylactic reaction (n = 1), possible, probable, or definite breakthrough fungal infection (n = 6), development of new neutropenic fevers (n = 2), elevated LFT's (n = 2), possible AMB associated bone marrow suppression (n = 1), and unknown (n = 4).

### Patients Treated in Both the AMB and AZ Periods

Twenty-seven patients, representing 82 hospitalizations, were treated in both periods. These patients were determined to have similar rates of renal dysfunction, hepatic dysfunction, and breakthrough fungal infections as those patients solely treated in a single study period.

### Cost Analysis

When the cost of the AZ period was compared to the AMB period, a $9,128/hospitalization increase in the mean total cost, $9,390/hospitalization increase in the mean pharmacy costs, and $68/hospitalization increase in mean laboratory costs was observed (Tables 3, 4). Multivariable analyses confirmed significantly less cost associated with AMB period (95% CI 0.65 – 0.78; p < 0.0001).

**Table 3:** Hospital costs (US Dollars)*

	AMB Period vs. AZ Period			AZ Period	
	AMB Period (n = 259)	AZ Period (n = 321)	p-value^1^	FLU (n = 237)	VOR (n = 84)
Mean Total	$44,129	$53,257	0.0141	$42,350	$66,975
Costs^2^	($39,643–$49,122)	($47,834–$59,296)		($37,952–$47,257)	($55,892–$80,255)
Mean Pharm	$10,361	$19,751	<0.0001	$12,009	$32,484
Costs^2^	($9,043–$11,871)	($17,229–$22,642)		($10,447–$13,805)	($25,792–$40,911)
Mean Lab	$438	$506	0.02	$467	$548
Costs^2^	($402–$477)	($464–$552)		($427–$510)	($473–$636)

^1^p-values are from mixed effects logistic regression models that controlled for the number of hospitalizations. P-values reflect the comparison of the cost in the AZ period relative to the cost in the AMB period

^2^Reported means are least squared means from mixed effects regression models that controlled for the number of hospitalizations

All costs are rounded to the nearest dollar. All costs are means (lower 95% confidence interval to upper 95% confidence interval)

Note: AMB = Amphotericn; AZ = Azole; FLU = flunconazole; VOR = voriconazole; Pharm = pharmacy; Lab = Laboratory.

**Table 4:** Total, median, and mean acquisition cost of antifungals (US Dollars)

	AMB Period (n = 255)^1^				AZ Period (n = 317)^1^			
	n^2^	Total Cost^3^	Median Cost^4^	Mean Cost^4^	n^2^	Total Cost^3^	Median Cost^4^	Mean Cost^4^
AMB	254	$119,194	$70 ($65–$76)	$467 ($274–$661)	198	$36,129	$18 ($9–$27)	$113 ($40–$186)
FLU	33	$8,343	0	$29 ($2–$56)	228	$78,849	$173 ($108–$224)	$245 ($211–$278)
VOR	4	$10,485	0	$34 ($0–$76)	160	$216,559	$75 (0–$250)	$682 ($563–$802)
CASPO	7	$17,228	0	$66 ($11–$122)	43	$174,718	0	$537 ($333–$741)
ITRA	50	$7,984	0	$29 ($19–$40)	6	$833	0	$3 ($0–$5)
Total	348	$164,405	$70	$630	645	$507,175	$266	$1,577

^1^Number of hospitalizations with available charges

^2^Number of hospitalizations with charges for the individual antifungals

^3^All costs are rounded to the nearest dollar

^4^Median or mean cost (lower 95% confidence interval to upper 95% confidence interval). Reported means are least squared means from mixed effects regression models that controlled for the number of hospitalizations

Note: AMB = amphotericin; AZ = Azole; FLU = fluconazole; VOR = voriconazole; CASPO = caspofungin; ITRA = itraconazole

Of note, caspofungin, an expensive agent by acquisition cost, was available solely during the AZ period. When the cost of caspofungin was removed from the analysis for the AZ period, the mean increase in total cost and pharmacy cost decreased to $8,916 and $9,090, respectively. While the increase in pharmacy costs was substantial between periods, the increase in mean acquisition cost of antifungals increased by only $947/hospitalization during the AZ period. In addition to the increased cost associated with FLU and VOR, a $157,490 increased total cost associated with greater use of caspofungin was observed during the AZ period. When this cost of caspofungin was removed from the analysis, the increased mean antifungal cost of the AZ period further decreased to $476/hospitalization. Of note, severe renal dysfunction was associated with a $20,465 (95% CI $10,458 to $33,729; p < 0.0004) increased total cost and $5,973 (95% CI $2199 to $11,400; p = 0.03) increased pharmacy cost per hospitalization. Compared to those patients without hepatic toxicity, patients with severe hepatic toxicity had an associated increased total hospital cost of $35,543 (95% CI $30,409, to $41,499; p < 0.0001) and increased pharmacy cost of $12,411 (95% CI $10,178 to $15,111; p < 0.0001).

## Discussion

The current study was designed to compare patient outcomes and costs associated with AZ or AMB prophylaxis in NHO patients. While retrospective in design, the current investigation is the largest with respect to the evaluation of VOR for primary prophylaxis in NHO patients.

There are several limitations to the study. As with any retrospective, observational cohort study, the influence of confounding factors is a concern. An additional challenge was analyzing those patients who received multiple courses of antifungals. Using mixed or random effects logistic regression models that controlled for the number of hospitalizations and includes fixed effects for each medication and each period, this bias was statistically minimized. The subgroup analyses of those patients with multiple admissions, however, suggest that multiple hospitalizations did not correlate with worse outcomes.

Not surprisingly, the incidence of mild/moderate and severe renal dysfunction was significantly higher in the AMB period, consistent with other reports [1,8]. Notably, renal dysfunction was associated with significantly increased costs, independent of the study period.

While the nephrotoxic potential of conventional amphotericin is well recognized, the need for hemodialysis was found to be infrequent.

The rate of severe hepatic toxicity was not significantly different between the AMB and AZ periods. However, patients treated with VOR had a significantly greater rate of severe hepatic toxicity. Our findings regarding hepatic toxicity associated with VOR differ from those reported in previous prospective, controlled clinical studies; it is possible that the increased rate may be due to confounding factors, including concomitant use of hepatotoxic agents [1-4,7]. Nevertheless, VOR was associated with increased rate and severity of liver dysfunction.

While not statistically significant, the incidence of probable and definite breakthrough fungal infections was greater during the AMB period. However, it is interesting to note that all breakthrough Candida infections and 4 of the 5 cases of fatal possible breakthrough Aspergillus infections occurred in those patients who received FLU prophylaxis.

The AZ period was associated with an increase of >$9,000/hospitalization in the mean total and pharmacy costs. These increases in costs may represent the increased rate of allogeneic transplants conducted in significantly older patients treated during the AZ period. More recent improvement in supportive care medications have allowed for increased allogeneic and autologous transplantation in an older population of patients compared to the past. Despite the substantial increase in hospitalization costs, the increase in costs directly associated with antifungal acquisition represented a small percentage of this increase in cost. Costs included in this study do not reflect the charges or the actual reimbursement associated with any of the costs. It should be noted the calculated cost to reimbursement ratio was similar during the two time periods (AZ: 1.196 and AMB: 1.153).

## Conclusion

While an AZ-based prophylactic regimen is associated with increased cost, the reduced rate of nephrotoxicity and availability of oral dosage forms, suggests they be used preferentially over AMB. While the triazoles may offer certain advantages, an increased rate of hepatic toxicity may be associated with VOR when compared with AMB or FLU.

## Competing interests

The authors declare that they have no competing interests. Intramural departmental funds were used toward the financial support of the study.

## Authors' contributions

AR participated in the conception, design, and coordination of the study, carried out the acquisition, analysis, and interpretation of data, and drafted and revised the manuscript. LC participated in the conception and design, and carried out the initial acquisition, analysis, and interpretation of data. JI participated in the acquisition, analysis, and interpretation of data. CY participated in the acquisition, analysis, and interpretation of data. TM participated in the analysis and interpretation of data, and was involved in the drafting and revising of the manuscript. BJG conceived of the study, participated in its design and coordination, carried out the analysis and interpretation of data and drafted, and revised the manuscript. All authors read and approved the final manuscript.

## Pre-publication history

The pre-publication history for this paper can be accessed here:


